# Pathogenic Mechanisms of Idiopathic Nonallergic Rhinitis

**DOI:** 10.1097/WOX.0b013e3181aadb16

**Published:** 2009-06-15

**Authors:** James N Baraniuk

**Affiliations:** 1 Division of Rheumatology, Immunology and Allergy, Georgetown University, Washington, DC

**Keywords:** entopy, nociceptive nerves, autonomic dysfunction, chronic fatigue syndrome

## Abstract

Idiopathic nonallergic rhinitis (iNAR) has been difficult to define because of the long differential diagnosis of rhinopathy in the absence of allergic rhinitis. iNAR has traditionally been a diagnosis of exclusion with no clear unifying pathophysiology. Increased sensitivity to triggers such has climate changes, cold air, tobacco smoke, strong odors, and perfumes have been thought to be characteristic, but recent studies do not support this hypersensitivity hypothesis. New investigations of the local nasal environment and systemic "functional" syndromes have offered new insights into this condition. iNAR may be a heterogenous disorder that includes (1) anatomic abnormalities requiring nasal endoscopy for diagnosis, (2) incipient, *local *atopy (entopy), (3) dysfunction of nociceptive nerve sensor and ion channel proteins, and (4) autonomic dysfunction as found in chronic fatigue syndrome and other functional disorders.

## Introduction

Idiopathic nonallergic rhinitis (iNAR) is the diagnosis that remains after inflammatory, eosinophilic, atopic, infectious, drug, endocrine, and structural etiologies have been excluded. iNAR subjects have been presumed to have sensitivity to inhaled irritants, and symptom complexes polarized to either (a) sneezing and drip or (b) congestion. Published evidence suggests that iNAR is a heterogenous disorder that includes (1) anatomic abnormalities requiring nasal endoscopy for diagnosis, (2) incipient, "endogenous" atopy (entopy), (3) nociceptive nerve dysfunction, and (4) autonomic dysfunction as found in chronic fatigue syndrome and other systemic "functional" syndromes.

## Anatomy as destiny

"Anatomy as destiny" may hold for people with nasal deformities and complaints but negative allergy skin tests. Deviated nasal septums are common findings. The deviations may cause narrowing of the middle or inferior turbinate on the side of the deviation, and hypertrophy of the turbinates into the space vacated on the contralateral side. In general, rhinitis symptoms are proportional to the degree of list of the septum [1]. Collapse of the nasal tip and fleshy swelling of the alae nasi can obstruct the anterior nasal valve and increase nasal airflow resistance and is more prevalent in the elderly [2]. However, not all anatomic changes cause symptoms. Magnetic resonance imaging and CT scans taken to evaluate neurologic or orbital disease (n ~ 3000) revealed high rates for mucosal thickening > 4 mm (10%-15%), asymptomatic air-fluid levels (2.8%-4.6%), and even opacification of 1 sinus (2.9%-3.8%) [3-6]. McAuliffe et al proposed that nasal turbinate mucosal contact points were responsible for referred pain [7]. Although still a popular notion, retesting at these points did not confirm any relationship to either pain or rhinopathy [8,9].

Turbinate hypertrophy is a frequent cause of nasal obstruction leading to turbinectomy or other surgical procedures. However, very little is known about the pathophysiology of this condition [10]. Hypertrophic turbinate tissue is often used as a "normal" control for studies of nasal polyps. This is inappropriate because turbinate histology depends on the patient pathology [11]. Septal deviation with compensatory hypertrophy of the inferior turbinate showed normal glands with some fibrotic areas around vessels when compared with normal tissues. Perennial allergic rhinitis tissue had glandular hypertrophy and interstitial edema. Glandular hypertrophy is also found in chronic rhinosinusitis without nasal polyps [12]. The excessive secreted mucus may cause postnasal drip with throat-clearing cough or thick, tenacious anterior discharge. This condition will not respond to vasoconstrictors. The most striking changes were found in "vasomotor rhinitis" where there was a decrease in the size of glands and fibrosis of the lamina propria [11]. This study requires prospective confirmation using patients who have been carefully evaluated preoperatively. Without similar evaluations, studies using "hypertrophic turbinates" to represent the "normal" state must be viewed with caution. Intranasal steroids were able to decrease turbinate hypertrophy measured by CT scan [13]. The tissue structure(s) that were reduced in size were not identifiable by imaging.

Anatomic abnormalities should be excluded by nasal endoscopy.

## Entopy

Entopy refers to localized nasal allergy without systemic evidence of atopy [14]. Cutaneous and blood allergy tests are negative and eosinophilia is absent. However, subjects have intermittent "seasonal" (seasonal nonallergic rhinitis, SNAR) or "persistent" (persistent nonallergic rhinitis, PNAR) symptoms that suggest waxing and waning allergic rhinitis. Previously reported glucocorticoid-responsive iNAR subjects may also have had entopy [15].

Evidence of allergic inflammatory cells would support the concept of entopy. Previous nasal biopsy studies of very strictly defined perennial nonallergic rhinitis subjects found no significant elevations of nasal mucosal lymphocytes, antigen-presenting cells, eosinophils, macrophages, monocytes, mast cells, and other immunoglobulin E (IgE)-positive cells between iNAR patients and nonrhinitic controls [16,17]. Inflammation was unlikely because virtually all referred patients had been previously treated with intranasal glucocorti-coids [18,19]. Only 2 of 65 had nasal eosinophilia.

In contrast, Powe et al were able to identify immunohistological differences between perennial allergic rhinitis, nonallergic rhinitis, and nonrhinitic control subjects [20]. They used full length inferior nasal turbinate resection tissue and so may have been able to include patches of densely clustered inflammatory leukocytes in their cell density measurements. PNAR had sig-nificantly higher densities of total (CD3^+^), activated (CD25^+^), and allergen-naive (CD45RA^+^) T lymphocytes in their nasal mucosa (*P *< 0.025) than normal controls. However, CD4^+ ^and other lymphocytes had equivalent numbers in IR and PAR that were higher than those for controls. Some of these lymphocytes may have distinct T regulatory, interleukin-17, and other phenotypes. This remains to be explored. IR had significantly more CD8^+ ^cells than PAR (*P *= 0.02) and control subjects. PAR subjects had significantly greater epithelial HLA-DR*α*+ cell staining than IR (*P *= 0.007). Mucosal mast cells were elevated in both PAR and IR groups. Submucosal mast cells were positively correlated with CD45RA^+ ^cells in PAR (*P *= 0.03). In contrast, these mast cells were positively correlated with CD8^+ ^cells in IR (*P *= 0.02). Other studies that have measured tryptase and histamine in nasal lavage fluids probably lacked the sensitivity to identify the small magnitude of mast cell changes that may be present in IR. These studies require follow-up with quantitative reverse transcriptase-polymerase chain reaction, in situ hybridization, and other advanced investigations to determine whether there is a subtle but potentially pathogenic increase in mast cell populations in IR.

Nasal lavage fluid contained low levels of IgE to *Dermatophagoides pteronyssinus *in 22% of 50 PNAR subjects [21]. Nasal provocation tests with *Der p 1 *were positive by acoustic rhinometry in 54% of this PNAR cohort and 100% of *Der p 1 *positive skin test PAR subjects. Nasal lavage fluid from PNAR subjects contained 6.0 ± 5.0 *μ*g/L eosinophil cationic protein, which was significantly higher than that in nonrhinitic controls (2.1 ± 2.2 *μ*g/L). PAR subjects had the highest levels (15.0 ± 17.0 *μ*g/L). The profiles of CD4^+ ^and other leukocyte subsets were equivalent for PNAR and PAR after challenge, and distinct from nonrhinitic controls. Both immediate and dual immediate plus late-phase responses were identified after grass pollen extract in 22/35 PNAR subjects [22]. Nasal IgE to grass was detected in 35% of the PNAR subjects with positive nasal provocations, suggesting that the provocation may be more sensitive an indicator than a radioallergosorbent test or similar IgE tests in lavage fluid. These studies suggest that 22% to 63% of PNAR subjects from this population had entopy.

Other studies have not supported the entopy concept. Provocations with multiple glycerinated extracts identified 4/20 who responded to glycerin alone and so were disqualified as placebo responders [23]. Alternatively, these 4 may have had nociceptive hyperresponsiveness or multiple chemical sensitivity. Eleven of 20 patients had negative nasal challenges. Only 5 had positive challenges defined by total symptom scores. However, repeat provocations were all negative. These results were in contrast to those of Powe et al and Rondon et al,[20-22] who used single allergens in aqueous solutions rather than glycerinated stocks. Acoustic rhinometry may be a more sensitive and objective outcome than total symptom scores and peak nasal inspiratory flow rates, whereas nasal eosinophil counts would not be expected to change during the provocation.

Future studies may need to be stratified by positive allergen nasal provocation or allergen-specific IgE levels in nasal lavage fluid to determine if entopy is a significant mechanism in iNAR.

## Nociceptive dysfunction

The trigeminal nerve innervates the nasal mucosa through its first ophthalmic and second maxillary branch [24]. It contains fast conducting, myelinated type A*δ *and slow conducting nonmyelinated type C nerve fibers. The distal axons become highly branched. The deep venous sinusoids and arteriovenous anastomoses are richly innervated. The submucosal glands and their vessels have a network of fibers surrounding each acinus. Superficial lamina propria vessels are relatively poorly innervated. The very fine, smooth nerve endings are embedded in the tight junctions between epithelial cells. It is not known if these are derived from both A*δ *and C fibers or only C fibers.

Numerous sensations from the nasal mucosa chemosensory and mechanicosensory afferents are perceived at the cortical level. Rapidly transmitted (A*δ*) sensations include heat, burning pain ("First Pain"), and cold [25]. These afferents innervate brainstem systems that regulate the breath-to-breath work of breathing and rapidly activated systemic avoidance behaviors. Type C neurons convey the slower onset sensations of discomfort, paresthesia, and gentle touch. They may also convey the sensations of mechanical stretch caused by engorgement of venous sinusoid walls and changes in epithelial cell dimensions when osmotic conditions change in the epithelial lining fluid [26]. Specific classes of chemicals interact with distinct sensor proteins on epithelial cells and the nerve endings to induce the neural depolarization.

The collection of sensor proteins on a neuron may be in constant flux depending on the conditions of inhaled air, desensitization, inflammation, and neurotrophins. Neuroplasticity of sensors, other modulating receptors, and neurotransmitters is controlled in part by leukotriene B4, nerve growth factor and its *TrkA *receptor, brain-derived neurotrophic factor and neutrotro-phin-4 at the *TrkB *receptor, and neutrotrophin-3 at the *TrkC *receptor [27]. Mediators such as bradykinin and many other inflammatory peptides, histamine and other amines, purines (P2X receptors), protons (acid-sensing ion channels, ASIC), potassium ion channels, other leukotrienes, and arachidonic acid metabolites may act in concert on single neurons via "stimulatory autoreceptors". Combinations of these and transient receptor potential (TRP) ion channels may be present on other neurons [28]. One sensor combination that seems to be important for visceral innervation is the capsaicin receptor (TRP vanilloid 1; TRPV1), ASIC3, and P2X receptors [29,30].

**Figure 1:**
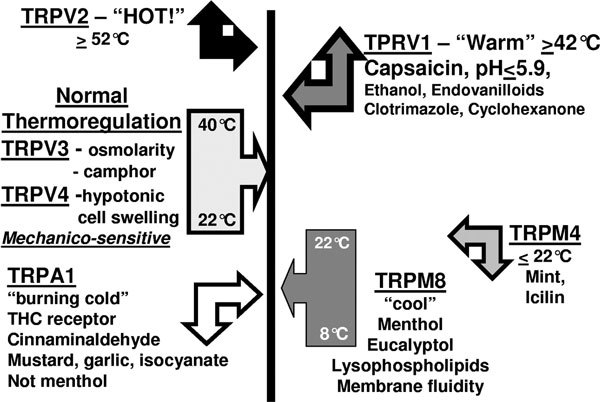
**TRP thermometer and aromatherapy**. The various TRP ion channels respond to different temperatures, chemicals, osmolarity, and other physical stimuli. The chemicals include many spices. The channels may respond to a large number of other chemical structures. Heterotetramers of these TRP proteins may vastly increase the variety of stimuli that can lead to trigeminal nerve depolarization.

At least 30 of the 143 TRP proteins may be present on nasal mucosal epithelial cells and neurons. These proteins form homotetramers and heterotetramers, which may finetune the precise conditions that activate them and lead to neuron depolarization. TRPV3 and TRPV4 are osmoreceptors that may respond to mechanical torsion applied by epithelial cells whose shapes change in response to variations in the tonicity of the epithelial lining fluid. Evaporation to humidify cold, dry air leads to a hypertonic fluid, whereas inhalation of steam with condensation on the mucosa may decrease the tonicity. These subtle changes may be evaluated by a "thermocouple" neuron by the changes in energy gained or lost as heat (enthalpy). TRPV3 and TRPV4 homotetramer-and heterotetramer-bearing neurons may convey messages of temperature between about 22°C and 40°C to the central nervous system (Figure 1).

TRPV1 is activated by capsaicin, ethanol, H^+^, local anesthetics, and temperatures above 42°C. Activation allows an influx of Ca^2+ ^and Na^+ ^that depolarizes the neuron. More dangerous temperatures above 52°C activate TRPV2 ion channels that depolarize A*δ *nerve fibers. Cool temperatures below 22°C activate TRP melanostatin 4 and TRPM8. TRPM4 is also the receptor for mint; its primary function is likely as a chemoreceptor. TRPM8 is the menthol receptor. A population of TRPM8^+ ^A*δ *nerve fibers are critical for assessing the breath-to-breath evaporation of water from the epithelial lining fluid and so the cooling of the superficial mucosa. Greater cooling implies faster airflow, more evaporation, a low resistance for airflow, and so nasal patency. Cooling also affects the mobility of lysophospholipids in the plasma membrane. This membrane fluidity is the probable regulator of TRPM8 activity in vivo [31]. The TRPM8 input to the brain stem helps determine the muscular force required to inhale air, and so the work of breathing. Dysfunction of this system may contribute to dyspnea. More extreme cold activates TRP ankryn 1 ion channels. TRPA1 is the receptor for garlic and mustard oil isocyanate compounds and tetrahydrocannabinol, but does not respond to menthol or mint.

Topical nasal capsaicin is an effective therapy in iNAR [32]. The capsaicin treatment reduced congestion for as long as 6 months. It had no effect in allergic rhinitis. These data suggest that increased nociceptive nerve function plays a role in iNAR, and that specific inactivation of TRPV1 has a role in its treatment.

Nasal neurons are generally polymodal, but some are relatively monomodal. Itch is mediated in part by a population of very thin, slow-conducting neurons that express the histamine H1 receptor [33]. Gastrin-releasing peptide (GRP) may be the neurotransmitter that relays primary itch messages to secondary spinothalamic projection interneurons in the spinal cord dorsal horn. Nasal neurons contain combinations of tachykinins (substance P and neurokinin A), the potent vasodilator calcitonin gene-related peptide (CGRP), glutamate, and purines [34]. Each combination is likely to convey a distinct message through the dorsal horn interneurons. The combinations are subject to change in response to inflammation, neurotrophins, and possibly other stimuli. These changes may occur as part of a chronic repatterning of dorsal horn connections that leads to hyperalgesia ("central sensitization") [25,35].

The function of type C and other types of nerves can be down-regulated by "inhibitory" autoreceptors that cause membrane hyperpolarization. These include adrenergic *β*2 and *α*2_C2_, histamine H3, *γ*-aminobutyric acid (GABA)_B_, serotonin HTD_3_, neuropeptide tyrosine Y2, and other G-protein coupled receptors [24]. These actions may be more important at central synapses and on efferent sympathetic and parasympathetic neurons. Additional classes of regulatory proteins may orchestrate depolarization, repolarization, and hyperpolarization. For example, leucine-rich pentatricopeptide motif containing protein is localized to the mitochondria of nociceptive nerves [36]. A mutant isoform causes the loss of mitochondrial cytochrome c oxidase activity followed by neurodegeneration. Comparable mutants may lead to peripheral and visceral neuropathies and to alterations in the neurotransmission of airway sensations such as "congestion", "rhinorrhea", "sinus pain and headache", and other iNAR symptoms.

When sensory neurons are stimulated, a wave of depolarization passes throughout the central axon and the extensively branched neural ramifications in the mucosa. This depolarization is maintained and transmitted by voltage-dependent sodium channels (Na(v)). The Na(v) family plays a role in pain transmission [37]. Genomic studies have linked mutation of the Na(v)1.1 protein to familial hemiplegic migraine headaches. A point mutation in Na(v)1.7 (F1449V) leads to "primary erythermalgia", a congenital disorder of severe pain and flushing [38]. Na(v)1.7 is expressed in dorsal root ganglia with Na(v)1.8. The mutant Na(v)1.7 causes a gain of function phenotype with hyperexcitability of nociceptive nerves [39]. In contrast, sympathetic neurons do not coexpress Na(v)1.8. In these neurons, the mutant Na(v)1.7 leads to a loss of function and decreased sympathetic activity. The selectivity of Na(v)1.8 for dorsal root ganglion cells may explain why its antagonists are highly selective analgesics. Pharmacological investigation of drugs such as these and TRPV1 antagonists on nociceptive nerve function in iNAR are eagerly awaited.

**Figure 2:**
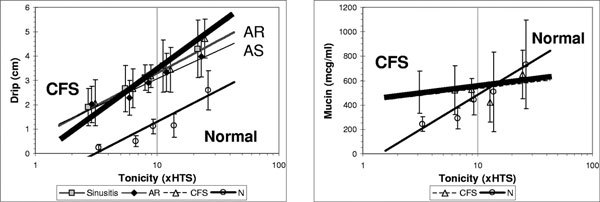
**Hypertonic saline nasal provocation responses in CFS (thick line), normal (thin dark line), AR, and AS**. A, Perceptions such as "Drip" (sensation of nasal discharge) were equivalent in CFS, AR, and AS and significantly higher than normal. B, Whereas the normal group had a significant dose response for axon response-mediated secretion of mucin, there was no dose dependence for the CFS group. AS indicates acute sinusitis.

The extensively branched neuronal processes in the nasal mucosa have efferent functions. They have swellings called varicosities that secrete neurotransmitters into the interstitial milieu. Release of CGRP leads to vasodilation, whereas neurokinin A, substance P, and GRP are glandular secretagogues. This efferent function of afferent type C nerves is the axon response, and the effects of the released neurotransmitters are referred to as neurogenic inflammation. In humans, unilateral hypertonic saline stimulates unilateral sensations of first and second pain, nasal congestion and rhinorrhea, the release of substance P into nasal lavage fluid, and glandular exocytosis [40]. There is no vascular component of either swelling or exudation. In contrast, "chronic fatigue syndrome" (CFS) subjects who have been found to have a high prevalence of a form of nonallergic rhinitis [41] have significantly different axon responses. Hypertonic saline nasal provocation causes exaggerated pain responses, but flat glandular secretory responses that are not dose-dependent (Figure 2) [42]. This indicates that the axon response, and so peripheral type C neuron function, is defective in this subset of iNAR.

CFS subjects also have greater tenderness of their sinus regions than normal controls, allergic rhinitis, and acute and chronic rhinosinusitis subjects [43]. This indicates systemic hyperalgesia in CFS, with light touch leading to the sensation of severe pain.

These findings implicate trigeminal hyperresponsiveness, hyperalgesia, and allodynia in this subtype of iNAR. Under normal circumstances, primary trigeminal type C afferent neurons synapse on spinothalamic projection neurons in the 2 superficial layers of the dorsal horn of the upper cervical spinal cord. A*δ *and A*β *fibers project to spinothalamic neurons in deeper laminae. This deeper set of interneurons also projects back to the superficial type C projection neurons and can increase their ability to convey pain messages. To prevent inappropriate neurotransmission of pain signals, these same type A afferents simultaneously stimulate interneurons that release GABA and inhibit the superficial projection neuron. This balance may be influenced by descending brain stem antinociceptive aminergic neurons that also inhibit the superficial neurons. Glial cells communicate with all of these cells.

Prolonged afferent activation, nerve injury, and neurotrophin generation lead to up-regulation of TRPV1 and probably other sensor proteins on the peripheral, mucosal nerve endings. These nerves become easier to depolarize in response to noxious and other stimuli. This state is termed "peripheral sensitization".

Peripheral sensitization leads to increased stimulation of the type C spinothalamic projection neurons. Easier transmission of pain impulses from type C neurons through these secondary neurons to cortical centers accounts for hyperalgesia. As the spinothalamic and other, regulatory interneurons become overstimulated from peripheral input, they adapt by becoming more efficient at transmitting the nociceptive input. This "central sensitization" represents spinal cord hyperexcitability. Peripheral light touch and potentially cold input from mechanoreceptive and other A*δ *fibers now become perceived as pain (allodynia). Presynaptic opioid receptors and calcium ion channels and postsynaptic sodium channels and receptors for glutamate, norepinephrine, 5-HT, and GABA are induced during central sensitization. Inhibitory interneurons and descending aminergic modulatory control systems become less effective, permitting disinhibition or facilitation of spinal cord dorsal horn neurons and worsening of the central sensitization. Glial cells participate by become activated and releasing cytokines and glutamate that decrease the threshold for depolarization (increase the excitability) of the spinothalamic projection neurons.

With this degree of dysfunction, minimal peripheral input of any type or even spinal cord neurons can begin to initiate autonomous messages of pain and discomfort analogous to phantom limb pain ("phantom nasal congestion"?). When localized to a single organ or limb, this state is referred to as a chronic regional pain syndrome (formerly, reflex sympathetic dystrophy). Generalization of this mechanism may lead to the widespread chronic pain of fibromyalgia and CFS. Dysfunction of the spinothalamic projection neurons may also have detrimental effects on the reticular and other brain stem systems leading to autonomic dysfunction and thalamic dysregulation of visceral and other sensory input.

This spinal cord reprogramming process is consistent with the dysfunctional rhinitis of CFS and the heightened trigeminal chemosensitivity found in subjects with sick-building syndrome and multiple-chemical sensitivity [44]. These supersensitive suffers may have survived natural selection by serving as vigilant sentinels using their heightened chemical sensitivity in the hunt for carion, mates, and the avoidance of predators and brush fires.

The influence of cold, dry air on airway functions is important for iNAR. Dose-dependent nasal airflow obstruction follows inhalation of cold, dry air only in nonallergic rhinitis. Dose dependence is absent in allergic rhinitis and nonrhinitic subjects [45]. This suggests a hyperresponsiveness of the cold-sensing neural afferents. The mechanism of the nasal obstruction is unclear, but is presumed to involve swelling of the deep venous sinusoids. Inhalation of histamine or bradykinin does not lead to dose-dependent airflow obstruction in iNAR [46]. Inhalation of cold, dry air with measurement of nasal airflow resistance or patency by acoustic rhinometry is currently the consensus standard for objective identification of iNAR [47].

Cold air leads to excessive nasal blockage with copious discharge in some skiers ("skier's rhinitis") [48]. The rhinorrhea is due to a hyperactive afferent--cholinergic parasympathetic reflex arc that can be effectively blocked with intranasal anticholinergic drugs. This reflex must be distinguished from the anterior discharge that is produced by the condensation of water from humidified air being exhaled through the nose.

Indoor exercise also involves heating, cooling, and humidifying inhaled air. Exercise caused rhinitis symptoms that adversely affected performance in 40% of subjects drawn from a community allergy practice and an exercise facility [49]. Allergic rhinitis subjects had more frequent symptoms both indoors (69% vs 53%; *P *= 0.04) and outdoors (72% vs 41%; *P *< 0.001) compared with nonallergic individuals. Anterior and posterior rhinorrhea was the most common single symptom, being present in half the symptomatic subjects. Nonallergic rhinitis subjects were not specifically identified in this population, but are implicated as those with negative allergy skin tests and positive responses to exercise. Aerobic exercise leads to potent vasoconstriction and a decongestant effect,[50] but is complicated after exercise by rebound rewarming of the mucosa with congestion and parasympathetic glandular secretion. This suggests that sympathetic nervous system hypofunction may also contribute to exercise-related rhinorrhea and other complaints. More focused studies will be required to investigate the mechanism(s) in allergic subjects who have exercise-induced symptoms ("mixed rhinitis") and iNAR subjects (negative allergy skin tests).

In addition to cold air, other climate conditions such as changes in temperature, humidity, barometric pressure, the passing of weather fronts through a region, and walking into or out of air-conditioned settings have been identified as potential triggers of iNAR symptoms. Other "consensus" triggers include cigarette smoke, consumption of alcoholic beverages, stressful or other emotional states, and strong perfumes and odors including cleaning fluids, photocopier toner, newsprint, and gasoline. Congestion and rhinorrhea are the critical elements for distinguishing between groups in classifications of "runners-sneezers" versus "blockers" and "wet" versus "dry" iNAR. "Dry" subjects have increased sensory nerve sensitivity to innocuous stimulation ("cacosmia"). Their complaints may represent allodynia with increased pain and discomfort. "Wet" nonallergic rhinitis is characterized by increased nasal congestion and copious discharge in response to secretagogues such as methacholine, nicotine, and capsaicin and nonspecific stimuli such as irritants, bright lights, and spicy foods ("gustatory rhinitis") [51,52]. In gustatory rhinitis, eating activates oral trigeminal receptors that recruit parasympathetic cholinergic reflexes and glandular secretion. At least 69% of survey respondents (396/571) had gustatory rhinitis symptoms to at least 1 food [53]. Foods ranged from hot chili peppers (49%) to bread (5%). Surprisingly, allergic rhinitis (*P *< 0.001) and a history of smoking (*P *= 0.049) were most highly correlated to gustatory rhinitis. This form of hyperresponsiveness may represent an increased sensitivity of sensory nerves to irritant stimuli with an increased parasympathetic reflex arc and augmented glandular responses. Curiously, denervation of rabbit sinus and nasal mucosa leads to glandular enlargement [54].

**Figure 3:**
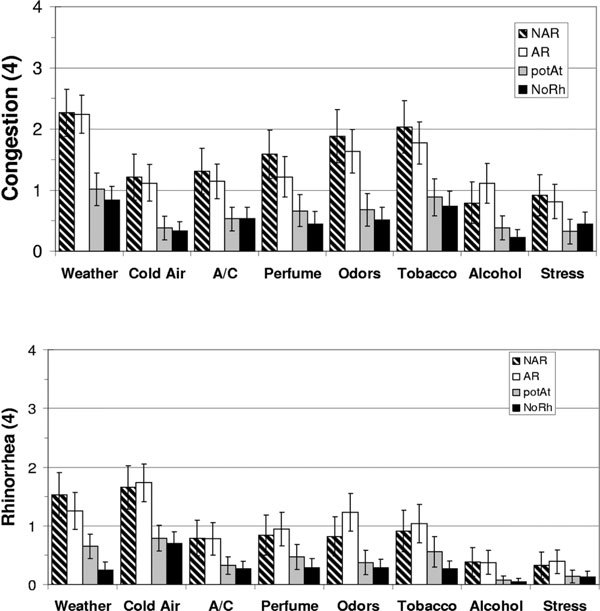
**Sensations of congestion (A) and rhinorrhea (B) induced by 8 triggers in NAR (bars with diagonal lines), AR (white bars), potAt (positive allergy skin tests with minimal symptoms; gray bars), and NoRh (black bars)**. Mean ± 95% CI. CI indicates confidence interval; NAR, nonallergic rhinitis; NoRh, no rhinitis; potAt, potential atopy.

We developed a questionnaire to determine which of these triggers provoked the most severe congestion and rhinorrhea [55]. For congestion, AR (allergic rhinitis) and iNAR subjects had much higher scores for weather conditions, tobacco smoke, and odors compared with subjects with low levels of rhinitis complaints (Figure 3) [56]. Weather and cold air generated the highest rhinorrhea scores. In essence, the level of rhinitis complaints, but not allergy skin test status, discriminated the high from the low congestion and rhinorrhea scores for each trigger. One interpretation is that allergy status is independent of the increased cortical perceptions of nasal congestion and discharge that are triggered by these inhaled stimuli. The responses of AR subjects to these "non-allergic" triggers may provide an explanation for so-called "mixed rhinitis". It remains to be determined if the sensor and neural mechanism(s) are the same in atopy and iNAR.

## Autonomic dysfunction

Autonomic dysfunction has long been associated with nonallergic rhinitis [57]. The relationship has been examined in prospective fashion in 78 subjects by Elsheikh and Badran [58]. Subjects' symptom scores were between 3 and 4 of 5 for rhinorrhea, nasal obstruction, and headache. Self-reported hyposmia (1.7/5) and sneezing (1.2/5) were less severe complaints. Although PNAR is often broken into "runners and sneezers" and "blockers", 71% had watery discharge and 29% had mucoid discharge (total 100%). Only 10% complained of sneezing. Obstruction was unilateral in 29%, raising the possibility that an anatomic abnormality may have been found on nasal endoscopy. Half of the subjects reported simultaneous, bilateral nasal obstruction. Headache may have had migraine-like qualities, or was consistent with the mid-facial pain syndrome [59].

Mid-facial pain shares many characteristics with tension-type headaches. Patients describe nasal pressure, heaviness, or tightness and may say that their nostrils feel blocked even though there is no obstruction to nasal airflow [60]. Regions of pain are generally symmetrical and affect the bridge of the nose, periorbital, and maxillary regions. Symptoms may begin as intermittent episodes and progress to a continuous ache. There are no clear exacerbating or relieving factors. Analgesics, antibiotics, and intranasal steroids are ineffective unless there is a strong, prolonged placebo effect. A 6-month course of low-dose amitriptylline (20 mg) has been shown to be beneficial. The mechanism and locus of action are unknown.

Lightheadedness was also common (67%) in dysautonomic rhinitis, although vertigo was not (17%) [58]. This symptom may have been more related to orthostatic hypotension and other cardiovascular and sympathetic nervous system complaints. Palpitations were present in 72%. Complaints of bronchospasm not confirmed as asthma (29%), gastroesophageal reflux (62%), and irritable bowel syndrome (88%) indicate that nonallergic rhinitis is only 1 part of a larger pattern of autonomic and other mucosal organ dysfunction syndromes. Other "allied" "functional" disorders where autonomic dysfunction plays a major role include CFS, fibro-myalgia (systemic hyperalgesia), Persian Gulf War Illness, migraines, and interstitial cystitis [61-64]. Impaired sympathetic reflexes have been demonstrated by heart rate variability,[65-67] neurally mediated hypotension on tilt table testing,[68] and impaired nasal vasoconstrictor responses to exercise, isometric muscle contraction, noise, and other stimuli [69-71].

The high prevalence of irritable bowel syndrome (IBS) in iNAR is of importance because of correlations of IBS subtypes with specific abnormalities in the enteric serotonin (5-HT) and dopamine systems and central pain, sympathetic autonomic, and parasympathetic autonomic nervous systems [72]. The molecular defects of these syndromes may provide insights into potential subsets of iNAR [73,74]. Potentiation of 5-HT release and activation of 5-HT1P or 5-HT3 receptors have been linked to diarrhea-predominant IBS. Conversely, reduced release of 5-HT, desensitization of 5-HT4 prokinetic receptors, and reduced dopaminergic function have been implicated in constipation-predominant IBS. The latter subtype may be related to the development of Parkinson's disease in later life [75]. Epidemiological relationships between iNAR and neurodegenerative diseases have not been examined. Visceral nociception is increased in IBS [76]. Intestinal smooth muscle dysmotility is common to IBS and disorders of bronchial, esophageal, bladder, and other smooth muscles [77-80]. These findings suggest shared mechanisms of dysfunctional peripheral, visceral, and central nociceptive afferent and efferent autonomonic neural systems for iNAR and these other "functional" disorders. The dysautonomia with blunted sympathetic responses to stressors and generalized elevation in parasympathetic influences may be the common denominator. Drugs that are active in these other illnesses may be useful for differentiating and treating subtypes of iNAR.

## Note

Supported by Public Health Service Awards 1 RO1 ES015382 and P50 DC006760 and Department of Defense Award W81XWH-07-1-0618.

Presented at a roundtable conference held in December 2008. The meeting was sponsored by the TREAT Foundation (Washington, DC) and supported through an unrestricted educational grant from Meda Pharmaceuticals. The funding company did not have any input into the development of the meeting or the proceedings, and the company was not represented at the roundtable meeting.
